# Key Determinants of SARS-CoV-2 Testing Among Symptomatic Individuals During the Second Wave in Uttar Pradesh, India: An Analysis From Two Districts

**DOI:** 10.7759/cureus.71784

**Published:** 2024-10-18

**Authors:** Raghukul R Pandey, Monika Agarwal, Brian Wahl, Tushar Garg, Amita Jain

**Affiliations:** 1 Department of Microbiology, King George's Medical University, Lucknow, IND; 2 Department of Community Medicine and Public Health, King George's Medical University, Lucknow, IND; 3 Yale School of Public Health, Yale University, New Haven, USA; 4 Global Health Campus, Stop TB Partnership, Geneva, CHE

**Keywords:** andersen's behavioral model, covid-19, healthcare access, logistic regression, multiple correspondence analysis (mca), psychological barriers, sars-cov-2, testing, uttar pradesh

## Abstract

Introduction: The COVID-19 epidemic caused significant disruptions worldwide, particularly in healthcare systems. India's second wave, driven by the Delta variant in 2021, severely affected healthcare capacity, leading to resource shortages and healthcare service disruptions. In this context, understanding the factors influencing SARS-CoV-2 testing is crucial for improving public health responses. This study investigates testing determinants in Uttar Pradesh, India, using Andersen's Behavioral Model of Health Services Use.

Methodology: We chose Lucknow and Sitapur districts in Uttar Pradesh based on the number of SARS-CoV-2 tests conducted per million people during the second wave of the epidemic. We conducted a cross-sectional study and surveyed 675 consenting respondents aged 18 and above from both districts. These respondents reported experiencing at least three COVID-19 symptoms between March and June 2021 (the second wave in the state). The survey was conducted face-to-face using a structured questionnaire on an electronic device. We used multiple correspondence analysis (MCA) to identify underlying factors, which were then utilized in a logistic regression model to assess their impact on SARS-CoV-2 testing.

Results: The testing rate in Lucknow (281, 84.6%) was higher than in Sitapur (117, 34.1%) (*P* < 0.001). Urban residents had a higher likelihood of being tested (188, 75.8%) than rural residents (210, 49.2%) (*P* < 0.001). Males (213, 63.0%) were more frequently tested than females (185, 54.9%) (*P* = 0.032). Postgraduates had the highest testing rate (49, 89.1%) compared to those without formal education (73, 44.8%) (*P* < 0.001). Individuals in regular jobs were more likely to be tested (171, 67.1%) compared to homemakers (128, 51.2%) and laborers (72, 57.1%) (*P* = 0.004). Smaller households (<5 members) had higher testing rates (146, 69.9%) than larger ones (252, 54.1%) (*P* < 0.001). Those living closer to a facility were more frequently tested (90, 64.3%) compared to those farther away (61, 34.1%) (*P* < 0.001). Additionally, individuals with access to public transport had higher testing rates (294, 62.0%) compared to those without (104, 51.7%) (*P* = 0.013). Higher-income groups were more likely to be tested (14, 93.3%) than low-income individuals (39, 36.8%) (*P* < 0.001). Psychological factors such as ease of testing (285, 72.5%) vs. (71, 38.6%) and perceived likelihood of needing testing (312, 90.7%) vs. (78, 25.1%) were strong predictors (both *P* < 0.001). Logistic regression identified urban residency and education as key determinants (odds ratio [OR] = 2.00, *P* < 0.001).

Conclusions: This study identifies key sociodemographic, logistical, and psychological factors influencing SARS-CoV-2 testing during the second wave of COVID-19 in Uttar Pradesh, India. Addressing disparities in healthcare infrastructure, improving health literacy, and reducing psychological barriers are essential to enhancing public health responses in future pandemics. Expanding healthcare access in rural areas and targeted public health campaigns could help bridge the gap in testing utilization. Further research is needed to explore these factors longitudinally and in different regional contexts.

## Introduction

The World Health Organization (WHO) declared COVID-19 a pandemic in March 2020, leading to widespread disruptions in social, economic, and healthcare systems worldwide [1]. By the end of 2022, countries around the globe had experienced multiple epidemic waves, each presenting unique challenges in terms of healthcare capacity, public health responses, and societal impacts. The initial waves exposed gaps in pandemic preparedness, healthcare infrastructure, and the ability to scale testing and treatment capabilities rapidly [2]. High-income countries also faced significant challenges despite advanced healthcare systems, while low- and middle-income countries (LMICs) struggled with the problem of limited resources and infrastructure [3].

India experienced a particularly devastating second epidemic wave of COVID-19 beginning in early 2021. This epidemic wave was characterized by a rapid increase in cases, partly driven by the emergence of more transmissible virus variants, such as the Delta variant (B.1.617.2) [4]. There was a sharp increase in cases from March to May 2021, followed by a rapid decline after May 2021 [5]. This surge overburdened healthcare facilities, leading to shortages of hospital beds, oxygen, and critical medical supplies. For example, hospitals in major cities like Delhi and Mumbai reported running out of oxygen supplies and ICU beds, resulting in a crisis [6].

The behavioral model of health services use, initially proposed by Andersen (1968), provides a framework to understand how individuals access and utilize health services [7]. According to this, service utilization is influenced by three main factors: predisposing, enabling, and need factors. Predisposing factors are the sociodemographic characteristics influencing an individual's liking to seek healthcare. Enabling factors are the logistical aspects that facilitate access to healthcare services. Need factors refer to the perceived and evaluated necessity for healthcare service utilization. Previous research assessed the association between sociodemographic factors and health service utilization, i.e., SARS-CoV-2 testing, and indicated the importance of further exploration of the association between these factors of the model [8]. 

Studies from various parts of the world have highlighted how existing inequalities in health systems are exacerbated during crises. For instance, a study in the United States found that socioeconomic disparities significantly impacted access to SARS-CoV-2 testing and treatment [9]. Similar trends have been observed globally, with vulnerable populations often facing the greatest barriers to healthcare services during the pandemic [10]. Previous research has also indicated that area of residence, caste, income, media consumption, and preference for healthcare settings are significantly associated with the utilization of healthcare services [8].

This study examines whether predisposing, enabling, and need factors, constructed as latent variables through multiple correspondence analysis (MCA), influenced the odds of undergoing the SARS-CoV-2 test during the second wave of the COVID-19 epidemic. MCA has been successfully applied to visualize the relationship between categorical variables in many fields, such as the social sciences, marketing, health, psychology, genetics, etc. [11]. Finally, logistic regression was employed to assess the impact of these factors on the outcome of interest.

## Materials and methods

Study design

**Figure 1:**
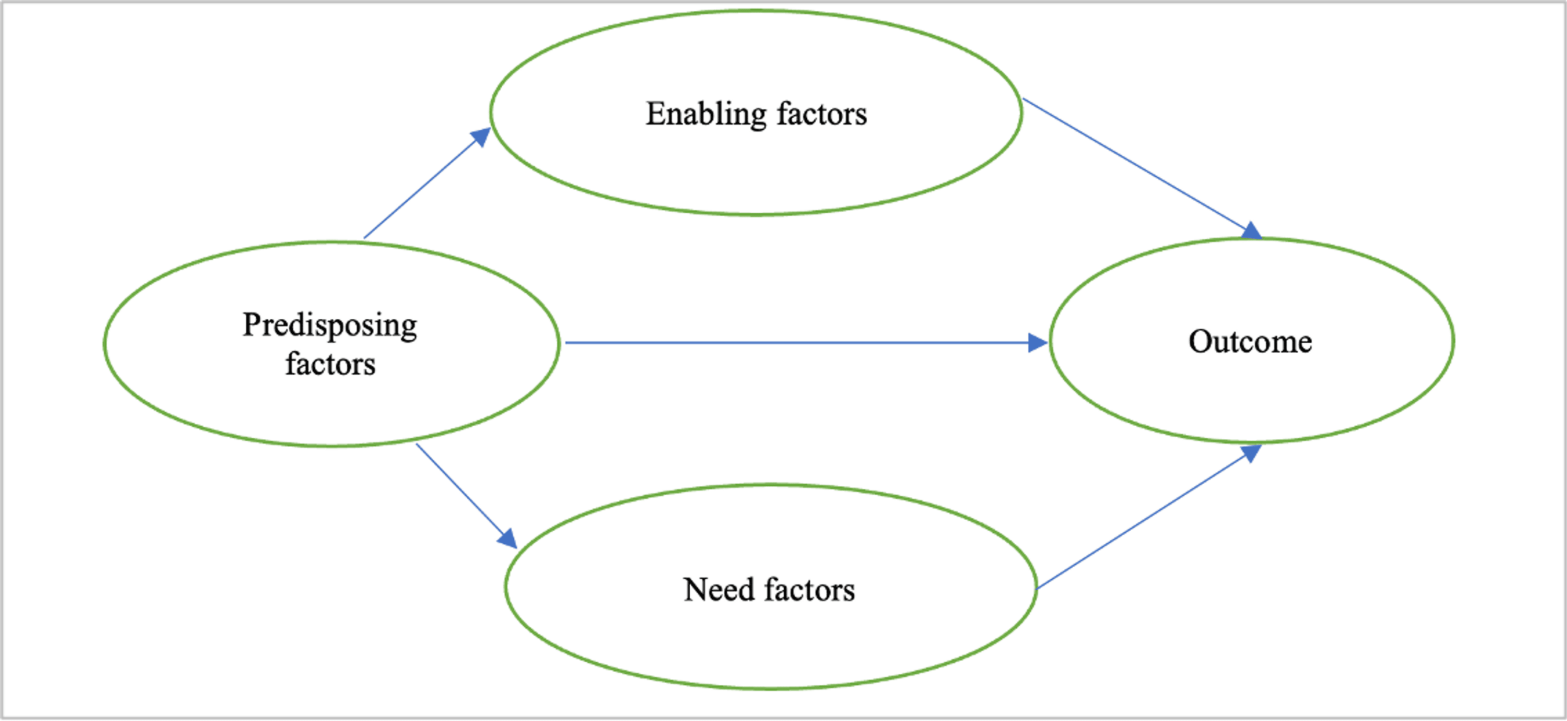
Conceptual model describing the relationship between predisposing, enabling, and need factors and outcome measured by whether a SARS-CoV-2 test was done when required in Uttar Pradesh during the second wave of the COVID-19 pandemic. Image credit: All authors.

We used a conceptual model to guide the study design and explore the relationship between predisposing, enabling, and need factors as determinants of health service utilization, specifically for SARS-CoV-2 testing (Figure 1).

We conducted a cross-sectional study from July to October 2023 in these two districts of UP selected based on reported maximum and minimum TPM during the second wave of the COVID-19 pandemic. The second wave in UP occurred between March and June 2021. This period was selected to assess the impact of the acute phase on testing demand and access in the contrasting urban and rural settings of the Lucknow and Sitapur Districts. 

Study setting

With an estimated population of more than 235 million, Uttar Pradesh (UP) is India's most populous state, accounting for approximately one-sixth of the country's population [12]. One of the major healthcare service providers, the Department of Medical, Health and Family Welfare, Government of UP, provides mainly primary and secondary healthcare services through more than 30,000 public health facilities, including 25,728 Health Subcenters, 3,645 Primary Health Centers (PHCs), 964 Community Health Centers (CHCs), and 107 District Hospitals to the people of the state, including 170 million people in rural areas [13].

We selected two state districts for our study based on the SARS-CoV-2 test per million (TPM) population reported from March to June 2021. District Sitapur reported the lowest TPM rate of 53,723 TPM, while district Lucknow reported the highest rate of 385,673 TPM in UP [14]. As per the latest available census of 2011, the population of both districts is almost similar (Lucknow, 4,589,838; Sitapur, 4,483,992), but the population density of Lucknow was more than three times (2528 persons/km^2^) compared to Sitapur (781 persons/km^2^) [12,15]. Lucknow is mainly urban, with (3,038,996, 66.2%) of the population living in urban areas, while Sitapur is predominantly a rural district, with only (530,784, 11.8%) living in urban areas. The proportion of literate persons was higher in Lucknow (3,127,260, 77.3%) compared to Sitapur (2,283,733, 61.1%) [16].

Study population

Our study included adults aged 18 years or above who resided in Lucknow or Sitapur District. To qualify for the study, participants had to have experienced at least three preidentified symptoms simultaneously indicative of SARS-CoV-2 infection between March and June 2021: (1) a new fever or a feeling of feverishness accompanied by chills or sweating, (2) a cough, and (3) mild or moderate difficulty in breathing, which could involve breathing faster than normal, difficulty fully inhaling or exhaling, or wheezing on exhalation. Additionally, participants were required to provide informed written consent signed in the presence of a witness. Participants were required to be physically and mentally capable of responding to our questions and understanding Hindi. Exclusion criteria included individuals under 18 years old, non-consenting individuals, pregnant or lactating mothers, anyone with a medical condition that might hinder effective communication, and residents of districts other than the selected districts.

Sample design

The sample size calculation for this study was guided by the principle of event per variable (EPV), specifically tailored to regression models with binary outcomes [17]. Following this principle, we used the following formula for the sample size:

 Sample size (*n*) = 100 + x*i

Here, *x* represents a predetermined integer value, and *i* denotes the number of independent variables planned for inclusion in the final regression model. To arrive at a reasonable sample size, *x* was fixed at 50, making the EPV formula effectively *n *= 100 + 50*i*. Anticipating the inclusion of at least ten independent variables into the final model, we computed the following sample size:

Sample size(n)=100+50×10=600

Factoring in a (60, 10%) nonresponse rate to accommodate potential dropouts or nonparticipation, the requisite sample size was adjusted to approximately 660.

We adopted a multistage cluster sampling technique to select study participants. After selecting two districts with the highest and lowest TPM, we further identified Community Development Blocks (CDB) and urban wards with the highest and lowest TPM within each of the two districts. By this, we selected two CDBs and two urban wards each from both districts. Then, we identified Primary Sampling Units (PSUs) from a list of census villages and Enumeration Blocks (EBs) in the selected CDBs and urban wards, respectively. The selection of the required number of villages and EBs was proportional to the urban and rural population distributions of districts. We choose PSUs based on Probability Proportional to Size (PPS) criteria [18], using the Census 2011 data as the sampling frame [19].

We further segmented each PSU into four equal parts after dividing the total number of households (HHs) in each village or EB, a process validated with the help of residents. We systematically selected five HHs reporting eligible individuals from each part through a circular random sampling method to choose around 20 HHs per village or EB. We selected one consenting Hindi-speaking eligible adult from each selected household. In cases where multiple eligible individuals lived in a household, we used the KISH grid to select the respondent. It is a grid for randomizing who to interview within a household when going door-to-door for data collection [20]. 

Data collection

We used Andersen's Behavioral Model of Health Services Use to design a structured questionnaire for data collection [7]. Initially, we developed the questionnaire in English and ensured the inclusion of relevant questions aligned with the study's objectives. The complete study questionnaire is provided in the Appendix. The questionnaire was later translated into Hindi using a direct and back translation approach. Before the study, we conducted a questionnaire pretest with 33 respondents from a population not involved in the main research. Then, the questionnaire was administered in Hindi, the region's local language. We collected the data from all eligible participants between July and October 2023 using Computer-Assisted Personal Interviews (CAPIs) for face-to-face data collection.

Data analysis

Our outcome variable, SARS-CoV-2 testing, is binary, with two possible responses: *Yes* or *No*. Most predictor variables are categorical, while a few continuous variables were transformed into categorical variables for analysis. A descriptive analysis was conducted to summarize the sociodemographic and behavioral characteristics of the study population. Then, bivariate analysis was performed to explore the relationships between these characteristics and outcome variables. Two statistical tests were applied depending on the characteristics of the data. Pearson Chi-Square test was used to explore the association between the categorical independent variables and SARS-CoV-2 testing. For variables with small cell counts (<5 in any category), Fisher's exact test was used to ensure the reliability of the results.

Finally, MCA was used to identify the underlying structure among the categorical variables in the dataset and to reduce the dimensionality of the data for regression analysis. MCA is suitable for handling and representing the relationships between categorical data [21]. The variables were grouped into predisposing, enabling, and need factors.

For predisposing factors, MCA was performed on variables including district, area type, age category, gender, religion, caste, marital status, education, occupation, individuals per household, media consumption habits like reading the newspaper, watching TV, and using the Internet on mobile phone, and source of healthcare in last five years. Dimension 1 accounted for (0.0475788, 67.1%) of the inertia, with the variables area, education, TV watching, and mobile internet use contributing more than 10%. These four variables were retained for further analysis, while the remaining were kept as individual predictors for the regression model. For enabling factors, MCA was applied to household income, distance to testing centers, and availability of public transport to testing centers. Dimension 1 captured the contribution of all enabling factors equally, so no variables were dropped from this group. For need factors, analysis was applied to the perception of COVID-19 symptoms, reliability of the test, the usefulness of the test in the prevention and treatment of COVID-19, nervousness about testing, feeling about testing positive for SARS-CoV-2, ease of testing, likelihood of getting tested, and perceived need for hospitalization. After removing the perception of COVID-19 symptoms variable (which did not contribute significantly), the remaining variables formed a meaningful structure for further analysis.

The MCA scores for predisposing, enabling, and need factors were predicted and used as constructs in the regression analysis. The score for predisposing factors was based on district, area type, education, TV watching, and mobile internet use; for enabling factors was based on household income, distance to testing centers, and public transport availability, while for need factors were based on the reliability of the test, the usefulness of the test in prevention and treatment of COVD-19. These scores were used as predictors in a logistic regression model where the dependent variable was whether an individual was tested for SARS-CoV-2. We tested multicollinearity using the Variance Inflation Factor (VIF) to ensure the regression model was robust. The results indicated there was no significant multicollinearity between the independent variables.

Finally, a logistic regression model was run with the dependent variable being SARS-CoV-2 testing status. The independent variables included the MCA scores for predisposing, enabling, and need factors, along with control variables such as age, gender, religion, caste, marital status, occupation, household size, a recent source of healthcare, perception about COVID-19 symptoms, testing nervousness, feeling about tested positive for SARS-CoV-2, ease of testing, and perception on getting tested. The model generated odds ratios (OR) for each predictor, allowing for an interpretation of the odds of testing based on different factors. Model fit statistics such as AIC and BIC were also calculated, and the goodness-of-fit test was performed to ensure the model was appropriate for the data. Data processing and statistical analyses were performed using Stata version 18 (Stata Corp. LLC, College Station, TX).

Human participation protection

We obtained approval from the Institutional Human Ethics Committee of King George's Medical University, Lucknow, UP, to conduct this study (Ref. code: 119th ECM II B-Ph.D./P1). Before initiating the interview, we obtained written informed consent from all the participants in the presence of a witness.

## Results

Table 1 reveals significant differences in SARS-CoV-2 testing rates across several characteristics related to demographic characteristics, i.e., predisposing factors. Individuals residing in district Lucknow were significantly more likely to have been tested (281, 84.6%) compared to those in Sitapur (117, 34.1%) (*P* < 0.001). Similarly, individuals from urban areas were more likely to get tested (188, 75.8%) compared to those from rural areas (210, 49.2%) (*P* < 0.001). Males had a higher testing rate (213, 63%) compared to females (185, 54.9%) (*P* = 0.032). Caste differences were significant, with Other Backward Caste (OBC) individuals being more likely to get tested (252, 63.8%) compared to those from General and Scheduled Caste/Tribes categories (*P *= 0.009). Higher educational levels were strongly associated with testing likelihood; postgraduates had the highest testing rate (49, 89.1%) (*P* < 0.001). Similarly, individuals engaged in regular earning jobs were significantly more likely to be tested (27, 67.1%) than homemakers and laborers (*P* = 0.004). Household size also played a role, with smaller households (<5 individuals) having a higher testing rate (146, 69.9%) compared to larger households (*P* < 0.001).

**Table 1:** Demographic characteristics of total and tested population for SARS-CoV-2 testing during the second wave in the two districts of Uttar Pradesh. 1. The chi-square statistic is derived from the observed and expected frequencies under the null hypothesis of no association. *P*-values indicate the probability of observing such associations by chance. A *P*-value less than 0.05 is considered statistically significant. 2. The demographic characteristics of the total population such as age category, gender, religion, caste, marital status, education, occupation, individuals per household, watching TV, using the internet on a mobile phone, and healthcare source in the last five years were reported in a previous paper [8].

SN	Characteristics	Particulars	Total population, *N* = 675 (%)	Tested population, *n* = 398 (% of total sample)	Pearson chi-square value	*P*-value
1	District	Lucknow	332 (49.2%)	281 (84.6%)	178.0055	<0.001
		Sitapur	343 (50.8%)	117 (34.1%)		
2	Area type	Rural	427 (63.3%)	210 (49.2%)	45.9660	<0.001
		Urban	248 (36.7%)	188 (75.8%)		
3	Gender	Male	338 (50.1%)	213 (63.0%)	4.6001	0.032
		Female	337 (49.9%)	185 (54.9%)		
4	Age category	18-39	332 (49.2%)	184 (55.4%)	4.3547	0.113
		40-59	275 (40.7%)	168 (61.1%)		
		60+	68 (10.1%)	46 (67.7%)		
5	Religion	Hindu	623 (92.3%)	366 (58.8%)	0.0032	0.955
		Muslim	48 (7.1%)	28 (58.3%)		
6	Caste	General	172 (25.5%)	92 (53.5%)	9.5316	0.009
		Other Backward Caste	395 (58.5%)	252 (63.8%)		
		Scheduled caste/ Scheduled Tribes	108 (16.0%)	54 (50.0%)		
7	Marital Status	Married	560 (83.0%)	335 (59.8%)	2.0729	0.355
		Never married	86 (12.7%)	49 (57.0%)		
		Widowed	26 (3.9%)	12 (46.2%)		
8	Education	No education	163 (24.1%)	73 (44.8%)	51.5182	<0.001
		Primary education	68 (10.1%)	34 (50.0%)		
		Secondary education	263 (39.0%)	147 (55.9%)		
		Graduate	126 (18.7%)	95 (75.4%)		
		Postgraduate	55 (8.1%)	49 (89.1%)		
9	Occupation	Homemaker	250 (36.0%)	128 (51.2%)	13.4111	0.004
		Regular earning	255 (37.8%)	171 (67.1%)		
		Student	44 (6.5%)	27 (61.4%)		
		Labor	126 (18.7%)	72 (57.1%)		
10	Individuals per household	< 5 individuals	209 (31.0%)	146 (69.9%)	14.8472	<0.001
		> 5 individuals	466 (69.0%)	252 (54.1%)		
11	Healthcare source in the last five years	Government	272 (40.3%)	174 (64.0%)	7.9090	0.019
		Private	388 (57.5%)	219 (56.4%)		
		Traditional Healers	15 (2.2%)	5 (33.3%)		
12	Reading newspaper	Almost every day	241 (35.7%)	203 (84.2%)	98.9158	<0.001
		Mostly not	434 (64.3%)	195 (44.9%)		
13	Watching TV	Almost every day	391 (57.9%)	285 (72.9%)	74.4948	<0.001
		Mostly not	284 (42.1%)	113 (39.8%)		
14	Using the Internet on a mobile phone	Almost every day	374 (55.4%)	277 (74.1%)	79.0455	<0.001
		Mostly not	301 (44.6%)	121 (40.2%)		

Table 2 shows that income and access to testing were critical testing determinants under enabling factors. Higher-income individuals were far more likely to have been tested (14, 93.3%) compared to lowest-income individuals (39, 36.8%) (*P* < 0.001). Similarly, proximity to testing canters influenced testing rates, with those living within 1 km of a testing facility having higher testing rates (90, 64.3%) than those living more than 5 km away (61, 34.1%) (*P* < 0.001). The availability of public transport was also a significant enabler of testing, with 294 (62.0%) individuals who had access to public transport being tested, compared to 104 (51.7%) of those without access (*P* = 0.013). Furthermore, under the need factor, the ease of the testing process emerged as a significant factor, with individuals who found the process easy being far more likely to have been tested (285, 72.5%) compared to those who found it difficult (71, 38.6%) (*P* < 0.001). The perception of the likelihood of getting tested was a strong predictor, with 312 (90.7%) of those who felt it was likely to get tested doing so, compared to only 78 (25.1%) of those who thought it was unlikely (*P* < 0.001).

**Table 2:** Comparison of Enabling, and Need Factors Between Total and Tested Population for SARS-CoV-2 Testing During the Second Wave in the two districts of Uttar Pradesh *Fisher's exact test *P*-values are reported where cell sizes are smaller than 5. A *P*-value less than 0.05 is considered statistically significant. For the remaining characteristics, Pearson's chi-square test is used. 1. Previous research reported the average household income in 2021 [8].

SN	Characteristics	Particulars	Total population, *N* = 675 (%)	Tested population, *n* = 398 (% of total sample)	Pearson chi-square value	*P*-value
Enabling factors						
1	Average household income in 2021	Below Poverty Line	106 (15.7%)	39 (36.8%)	50.2720	<0.001
		Low Income	345 (51.1%)	191 (55.4%)		
		Middle Income	208 (30.8%)	154 (74.0%)		
		High Income	15 (2.2%)	14 (93.3%)		
2	Distance of testing facility	Equal or less than 1 km	140 (20.7%)	90 (64.3%)	63.4214	<0.001
		2 to 5 km	356 (52.7%)	247 (69.4%)		
		More than 5 km	179 (26.5%)	61 (34.1%)		
3	Availability of public transport to reach the testing facility	Yes	474 (70.2%)	294 (62.0%)	6.1694	0.013
		No	201 (29.8%)	104 (51.7%)		
Need factors						
1	Fever, cough, and mild or moderate difficulty in breathing were common symptoms of COVID-19	Agree	568 (84.1%)	335 (59.0%)	1.5270	0.466
		Neither agree nor disagree	44 (6.5%)	29 (65.9%)		
		Disagree	63 (9.3%)	34 (54.0%)		
2	SARS-CoV-2 test reliable	Agree	654 (96.9%)	388 (59.3%)	1.1974	0.550
		Neither agree nor disagree	10 (1.5%)	5 (50.0%)		
		Disagree	11 (1.6%)	5 (45.5%)		
3	The usefulness of the SARS-CoV-2 test in preventing the spread of infection	Useful	663 (98.2%)	390 (58.8%)	-	0.442*
		Neither useful nor useless	9 (1.3%)	5 (55.6%)		
		Useless	3 (0.4%)	3 (100.0%)		
4	The usefulness of SARS-CoV-2 test for treatment of COVID-19	Useful	668 (99.0%)	393 (58.8%)	-	0.499*
		Neither useful nor useless	4 (0.6%)	2 (50.0%)		
		Useless	3 (0.4%)	3 (100.0%)		
5	Nervousness at the thought of getting tested for COVID-19	Nervous	587 (87.0%)	347 (59.1%)	0.7364	0.692
		Neither nervous nor relaxed	29 (4.3%)	15 (51.7%)		
		Relaxed	59 (8.7%)	36 (61.0%)		
6	Feeling about getting a positive test result for COVID-19	Calm	64 (9.5%)	38 (59.4%)	0.0050	0.944
		Scared	611 (90.5%)	360 (59.0%)		
7	The entire process of the COVID-19 testing	Easy	393 (58.2%)	285 (72.5%)	71.9254	<0.001
		Neither easy nor difficult	98 (14.5%)	42 (42.9%)		
		Difficult	184 (27.3%)	71 (38.6%)		
8	Chances of getting the COVID-19 test done	Likely	344 (51.0%)	312 (90.7%)	293.7053	<0.001
		Neither likely nor unlikely	20 (3.0%)	8 (40.0%)		
		Unlikely	311 (46.0%)	78 (25.1%)		
9	Need for hospitalization	No	559 (82.8%)	323 (57.8%)	1.8757	0.171
		Yes	116 (17.2%)	75 (64.7%)		

MCA results in Table 3 show that among the predisposing factors, urban-rural differences (12.5%) and education levels (8.6%) significantly contribute to testing, with reading habits and TV-watching also playing substantial roles. Access to information via internet usage (8.9%) further highlights its importance in testing decisions. Among enabling factors, household income disparities (45.6%) and proximity to testing centers (34.1%) emerge as major contributors, along with public transport availability (25.3%). Finally, for need factors, test reliability (19.6%) and perceived usefulness of the test (16.7%) highlight the importance of confidence in testing, while nervousness (12.3%) reflects emotional barriers impacting decisions.

**Table 3:** Explained inertia and key category contributions to the dimensions of predisposing, enabling, and need factors for SARS-CoV-2 testing in Uttar Pradesh, based on multiple correspondence analysis.

Dimension	Inertia explained (%)	Cumulative inertia (%)	Key categories	Contribution to dimension 1 (%)	Contribution to dimension 2 (%)
Predisposing factors					
Dim 1	67.12	67.12	Area type (Urban, Rural)	12.5	3.5
Dim 2	7.29	74.4	Education (Graduate, No Education)	8.6	2
Dim 3	5.44	79.84	Reading newspaper	10	1.2
Dim 4	1.09	80.94	Watching TV	9.8	3
Dim 5	0.42	81.36	Internet usage	8.9	0.5
Enabling factors					
Dim 1	95	95	Household income (Low, Middle, High)	45.6	12.7
Dim 2	2.47	97.47	Testing distance	34.1	8.3
Dim 3	0.02	97.49	Public transport availability	25.3	10.2
Need factors					
Dim 1	62.13	62.13	Test reliability (Reliable, Unreliable)	19.6	5.2
Dim 2	18.33	80.46	Usefulness of test	16.7	7.1
Dim 3	0.03	80.49	Testing nervousness	12.3	4.9

In Table 4, the logistic regression results highlight several key factors influencing SARS-CoV-2 testing. Predisposing factors (OR = 2, *P* < 0.001) are a strong determinant of testing odds, while enabling factors (OR = 0.76, *P* = 0.082) show some influence but are not statistically significant. Age is a significant factor, with individuals aged 40-59 (OR = 1.84, *P* = 0.026) and 60+ years (OR = 2.49, *P* = 0.043) more likely to get tested. Caste also plays a role, with OBCs significantly more likely to get tested (OR = 2.88, *P* = 0.001). Ease of testing (OR = 2.01, *P* = 0.015) increases the odds of testing, while individuals perceiving a low chance of needing a test are significantly less likely to undergo testing (OR = 0.04, *P* < 0.001).

**Table 4:** Logistic regression results for factors associated with SARS-CoV-2 testing in Uttar Pradesh.

Variable	Odds ratio (OR)	95% confidence interval (CI)	*P*-value
Score for Predisposing Factors	2.00	1.40-2.86	<0.001
Score for Enabling Factors	0.76	0.56-1.04	0.082
Score for Need Factors	1.04	0.67-1.60	0.864
Age Category: 40-59	1.84	1.08-3.16	0.026
Age Category: 60+	2.49	1.03-6.01	0.043
Caste: Other Backward Caste	2.88	1.57-5.28	0.001
Caste: Scheduled Caste/Tribe	1.53	0.70-3.36	0.284
Occupation: Regular Earning	1.83	0.76-4.38	0.176
Healthcare: Private Facility	0.64	0.39-1.05	0.074
Testing Nervousness: Relaxed	0.68	0.18-2.51	0.563
Testing Embarrassing: Neutral	0.51	0.27-0.95	0.035
Testing Easiness: Easy	2.01	1.15-3.52	0.015
Testing Chance: Unlikely	0.04	0.02-0.07	<0.001

Further analysis demonstrates a good fit model, as indicated by a Pseudo R-squared of 0.466, meaning it explains approximately 46.6% of the variance in the likelihood of testing for SARS-CoV-2. The Akaike Information Criterion (AIC = 544.23) and Bayesian Information Criterion (BIC = 679.4) further suggest a well-balanced model, achieving a reasonable trade-off between model complexity and goodness of fit.

## Discussion

Interpretation of findings

This study provides insights into the determinants of SARS-CoV-2 testing during the second wave of the COVID-19 pandemic in UP, India. The pandemic emphasized existing healthcare access inequalities, a particularly pronounced challenge in India during its devastating second wave [6]. Using Andersen's behavioral model as a framework, our analysis demonstrates the influence of predisposing, enabling, and need factors on healthcare utilization, particularly in pandemic circumstances where rapid response is crucial [7]. In line with global observations, this study found that sociodemographic characteristics significantly influenced individuals' odds to seek healthcare services such as SARS-CoV-2 testing [22].

Among the predisposing factors, urban residents were more likely to access testing services than their rural counterparts. This disparity could be attributed to better healthcare infrastructure and information dissemination in urban areas [23]. Education level emerged as a strong predictor of healthcare utilization, with higher education levels correlating with increased testing rates, possibly due to better health literacy [24]. The enabling factors highlighted the critical role of logistical support in healthcare access. Distance to healthcare facilities and the availability of public transport were significant determinants of testing uptake, underscoring the need for well-distributed healthcare resources during a public health crisis [25]. Lastly, the need factors such as perceived test reliability and psychological barriers like testing nervousness and stigma associated with a positive test result played substantial roles. This aligns with findings from other low- and middle-income countries where stigma and fear associated with COVID-19 significantly deterred healthcare utilization [26].

Public health implications

This study offers important public health implications, particularly in the context of pandemic preparedness, healthcare access, and the delivery of equitable health services. The findings highlight significant differences in SARS-CoV-2 testing uptake between urban and rural areas. Urban residents were more likely to get tested due to better healthcare infrastructure, accessibility, and information dissemination in urban settings compared to rural areas. Rural populations often face challenges such as poor access to healthcare facilities, limited transportation, and lower health literacy, possibly contributing to lower testing rates. This shows the urgent need for targeted public health interventions to improve access to testing and healthcare services in rural areas, where infrastructure is often underdeveloped. Decentralizing healthcare resources by establishing more community health centers or mobile testing units in rural regions could help address these disparities [27].

Education emerged as a strong predictor of healthcare utilization, with individuals possessing higher education levels more likely to access SARS-CoV-2 testing. This highlights the importance of health literacy in promoting the uptake of health services. Public health strategies should focus on improving health literacy, particularly in less educated populations, by using simple and accessible language in health campaigns. This can ensure better understanding and engagement with public health measures [28]. The study also highlights the importance of logistical support, such as proximity to healthcare facilities and the availability of public transport, which were significant determinants of testing uptake. Addressing these logistical barriers is crucial for improving healthcare access, particularly in underserved areas. Psychological barriers like testing-related nervousness and stigma associated with a positive test result were found to play significant roles in testing decisions. Public health campaigns should address these barriers by normalizing testing and reducing the stigma surrounding COVID-19. Efforts such as public messaging from trusted sources and community engagement can help reduce fear and encourage testing [29,30].

Strengths and limitations

One of the key strengths of our study is its comprehensive analysis of SARS-CoV-2 testing determinants using a well-established framework - Andersen's behavioral model of health services use. The study utilizes robust statistical techniques, including MCA and logistic regression, to identify and quantify the impact of predisposing, enabling, and need factors on testing behavior. Another strength is its comparative approach, examining differences between urban and rural districts, highlighting significant disparities in healthcare access and utilization. Additionally, its large sample size enhances the reliability and generalizability of the findings within the context of UP, India.

Despite these strengths, the study has some limitations, so its findings must be interpreted within the context of inherent limitations. The study focuses on a specific health emergency (COVID-19) in a particular region, which may limit the applicability of the findings to other contexts or health crises. The cross-sectional design limits the ability to infer causality between the identified factors and testing behavior. The gap between the second wave and the study period, along with the reliance on self-reported data, may introduce recall bias, potentially affecting the accuracy of the findings. This could also lead to misclassification of testing status or symptoms, potentially biasing the results towards non-differential misclassification and diluting the proper associations. The exclusion of dead individuals due to COVID-19 from the study is likely to introduce survivorship bias, leading to an underrepresentation of the testing needs and barriers among the most vulnerable populations. The potential unmeasured confounders such as mobility and exposure risk, socio-cultural norms and beliefs around illness, healthcare-seeking behavior, and trust in the health system could significantly influence testing behavior, suggesting that the observed associations might not fully capture the complex interplay of factors affecting testing utilization. 

Recommendations for future research

Future research should focus on longitudinal studies to establish causal inferences regarding the evolving nature of healthcare-seeking behavior, especially during pandemics. Expanding the geographic scope of research to include diverse regions would enhance our understanding of disparities in healthcare access. Sociocultural influences and psychological barriers such as stigma and fear should be further explored using qualitative methods to understand community attitudes better. Additionally, the impact of policy interventions, such as mobile testing units and decentralized healthcare, needs to be evaluated for effectiveness in reducing disparities. Technological solutions like digital health tools and telemedicine could also be explored to promote equitable healthcare access. Finally, assessing the role of trust in healthcare systems will be critical in understanding and improving testing uptake and healthcare utilization during public health crises.

## Conclusions

This study provides valuable insights into the factors influencing SARS-CoV-2 testing behavior during the second wave of COVID-19 in UP, India, using Andersen's behavioral model as a framework. The findings reveal significant disparities in testing uptake between urban and rural populations, highlighting the critical role of healthcare infrastructure, sociodemographic factors, and logistical support in testing accessibility. Psychological barriers, such as stigma and nervousness, were also identified as significant deterrents, emphasizing the need for targeted public health interventions. While the study has limitations, including its cross-sectional design and potential recall bias, it offers a comprehensive understanding of the determinants of healthcare utilization in the context of a public health crisis.

## Appendices

Appendix

**Table 5:** Study questionnaire.

Identification Details	
District Name: Sitapur/Lucknow	District Code: Sitapur-1, Lucknow-2
Area: Urban/Rural	Setting Code: Urban-1, Rural-2
Block/Ward Name:	Block/Ward Code:
Gram Panchayat/mohalla Name:	Gram Panchayat/Enumeration block Code :
PSU Number:	
Respondent ID	
Date of Interview	Day Month Year
Start Time	Hour Minute
Result status of the questionnaire	Completed -1
	Partly Completed -2
	Not at Home 3
	Refused-4
	Postponed-5
	Others (Specify)-99

Section-1 Identification of symptomatic individuals during March-June 2021				
S.N.	Questions	Coding categories	Codes	Skip to
	Were you ever suffering from these new symptoms in the year 2021? INSTRUCTIONS: Multiple responses possible [Yes-1, No-2, Not sure-3] Please continue the interview only if the response to the first three coding categories is “Yes”. If the responses to the first three coding categories are “No”, please stop the interview and interview the next respondent.	New onset of fever or feeling feverish (such as chills, sweating)	1/2/3	
		Cough	1/2/3	
		Mild or moderate difficulty in breathing (breathing slightly faster than normal, feeling like you can’t inhale or exhale, or wheezing, especially during exhaling or breathing out)	1/2/3	
		Sore throat	1/2/3	
		Running or stuffy nose	1/2/3	
		Muscle or body aches	1/2/3	
		Headaches	1/2/3	
		Unusual fatigue	1/2/3	
		Diarrhea	1/2/3	
		New loss of Taste and/or smell	1/2/3	
		Nausea or vomiting	1/2/3	
	If yes, in which month did you suffer from these symptoms? INSTRUCTIONS: Multiple months are possible If the months do not include March, April, May or June or the respondent does not remember the name of the month, please stop the interview and interview the next respondent.	Name of the month		
		Do not know	98	

Section 2: Socio-economic and demographic details of symptomatic individuals during March-June 2021				
SN	Questions	Coding categories	Codes	Skip to
	How old were you on your last birthday?	Age in completed years		
	What is your gender?	Male	1	
		Female	2	
		Others (please specify)	99	
	What is your religion?	Hindu	1	
		Muslim	2	
		Sikh	3	
		Christian	4	
		Buddhist/neo-buddhist	5	
		Jain	6	
		Jewish	7	
		Parsi	8	
		Others (please specify)	99	
	What is your caste or tribe?	Scheduled caste	1	
		Scheduled tribe	2	
		Other backward caste	3	
		General	4	
		Others (please specify)	99	
	What was your marital status during March to June 2021?	Married	1	
		Divorced	2	
		Separated	3	
		Widowed	4	
		Never married	5	
		Widower	6	
		Don’t want to tell	98	
	Can you read and write?	Yes, can read and write	1	►110
		Yes, can read	2	
		No	3	
109.	What is the highest schooling standard that you have completed? INSTRUCTIONS: Code exact number of years of schooling.	Standard		
		Technical education after 10th Class	1	
		Graduate	2	
		Postgraduate	3	
		Others (please specify)	99	
	What was your main occupation before the start of the COVID-19 pandemic in 2020? (i.e., Work in which maximum time is given)	Self‐employed in agriculture/ fishery/orchard/animal husbandry	1	
		Self-employed in non-agriculture (like stitching, handy craft, business shop)	2	
		Regular salaried in government sector	3	
		Regular salaried in private sector	4	
		Daily wage labor	5	
		Casual wage labor in public works (e.g., MGNREGA)	6	
		Casual labor in agriculture	7	
		Casual labor in non‐agriculture other than public works	8	
		Traditional service occupation (Cobbler, Dhobi, Barber)	9	
		Unpaid family worker in agriculture/fishery/orchard/animal husbandry	10	
		Unpaid family worker in non-agriculture	11	
		Homemaker	12	
		Unemployed	13	
		Retired	14	
		Student	15	
		Other (please specify)	99	
	How has your occupational status changed after the first wave of the COVID-19 pandemic in 2020?	No Change occurred; everything is same as before	1	
		Occupation is lost and still searching for occupation	2	
		Occupation was lost but regained it before the second wave.	3	
		The occupation was lost but started a new occupation before the second wave.	4	
		Others (please specify)	99	
	What was your average monthly income? INSTRUCTIONS: Please mention the average income in a month.	During January-March 2020 Rs.		
		During January-March 2021 Rs.		
		Do not know	98	
	What was the average monthly income of all the members of your household? INSTRUCTIONS: Please mention the average income in a month.	During January-March 2020 Rs.		
		During January-March 2021 Rs.		
		Do not know	98	
	During the second wave, what was the total number of people living in your household?	1	1	
		2	2	
		3	3	
		4	4	
		5	5	
		More than 5	6	
	Do you read a newspaper?	Almost every day	1	
		Not at all	2	
		Sometimes	3	
	Do you watch television?	Almost every day	1	
		Not at all	2	
		Sometimes	3	
	Do you use internet on your mobile phone?	Almost every day	1	
		Not at all	2	
		Sometimes	3	
	Where have you been taking health services normally for the last 5 years?	Government hospitals & centers	1	
		Private hospitals & nursing homes	2	
		Qualified solo private doctor	3	
		Traditional healers	4	
		Others (please specify)	99	
	How many times did you get troubled by the following problems between March and June 2021? INSTRUCTIONS: Not at all-0, Several days-1, More than half a day-2, Nearly every day-3	Feeling nervous, anxious or on edge	0/1/2/3	
		Not being able to stop or control worrying	0/1/2/3	
		Feeling down, depressed, or hopeless	0/1/2/3	
		Little interest or pleasure in doing things	0/1/2/3	
	Have you been smoking cigarettes, bidis or any other tobacco products regularly for the last three years?	Yes	1	
		No	2	
		Do not want to tell	98	
	Are you suffering from any of the following diseases which are being treated for the last three years? INSTRUCTIONS: Multiple responses are possible. [Yes-1, No-2, Not sure-3]	Heart disease	1/2/3	
		Diabetes	1/2/3	
		Lung disease	1/2/3	
		Kidney disease	1/2/3	
		Cancer	1/2/3	
		Others (please specify)	99	
	Has any member of your house died between March 2021 to June 2021?	Yes	1	►127
		No		
		Do not know	98	
	Where did this death take place?	Government hospital	1	
		Private hospital	2	
		At home	3	
		During transportation	4	
		Other places (please specify)	99	
		Do not know	98	
	Was the deceased suffering from these new symptoms before death?	Fever	1/2/3	
		Cough	1/2/3	
		Mild or moderate difficulty in breathing	1/2/3	
	Whether the deceased was tested for COVID-19?	Yes	1	►127
		No	2	
		Do not know	98	
	What was the COVID-19 test result of diseased?	Positive	1	
		Negative	2	
		Equivocal	3	
		Repeat sample	4	
		Report was not received	5	
		Do not know	98	
Section 3: Factors associated with the access to and utilization of COVID-19 test				
	Do you agree that fever, cough and mild or moderate difficulty in breathing were symptoms related to COVID-19?	Strongly agree	1	
		Agree	2	
		Neither agree nor disagree	3	
		Disagree	4	
		Strongly disagree	5	
	When you were suffering from these symptoms, were you able to do your daily routine activities without any support?	Yes	1	
		No	2	
		Do not know	98	
	Did you need to be hospitalized?	Yes	1	►132
		No	2	
		Do not know	98	
	Did you have to be hospitalized?	Yes	1	►132
		No	2	
	If you needed to be hospitalised, why weren't you admitted to the hospital?	Could not get support of family members for hospitalization	1	
		Hospital with available beds not found	2	
		Fear of adverse outcome of hospital admission	3	
		Others (please specify)	99	
	Were people you knew getting tested when they were suffering from these symptoms?	Yes	1	
		No	2	
		Do not know	98	
	What was your view on the availability of government testing facilities for COVID-19? Instruction: Multiple responses are possible	It was available for everyone who needed it	1	
		It was available for symptomatic persons only	2	
		It was available to those who came in contact with confirmed cases	3	
		It was available for seriously ill patients only	4	
		It was available for rich and influential people only	5	
		Others (please specify)	99	
	How reliable was the COVID-19 test in your opinion?	Very reliable	1	
		Reliable	2	
		Neither reliable nor unreliable	3	
		Unreliable	4	
		Very unreliable	5	
	How useful was the COVID-19 test in knowing the level of infection?	Very useful	1	
		Useful	2	
		Neither useful nor useless	3	
		Useless	4	
		Totally useless	5	
	How useful was the COVID-19 test in preventing the spread of infection?	Very useful	1	
		Useful	2	
		Neither useful nor useless	3	
		Useless	4	
		Totally useless	5	
	How useful was the COVID-19 test for treatment?	Very useful	1	
		Useful	2	
		Neither useful nor useless	3	
		Useless	4	
		Totally useless	5	
	How nervous were you at the thought of getting tested for COVID-19?	Very nervous	1	
		Nervous	2	
		Neither nervous nor relaxed	3	
		Relaxed	4	
		Very relaxed	5	
	How embarrassing was it to get tested for COVID-19?	Very embarrassing	1	
		Embarrassing	2	
		Neither Embarrassing nor convenient	3	
		Convenient	4	
		Very convenient	5	
	Which samples were being taken for the COVID-19 test? Instruction: Multiple responses are possible	Blood	1	
		Urine	2	
		Stool	3	
		Cerebrospinal fluid (CSF)	4	
		Nasopharyngeal swab	5	
		Do not know	98	
	How is the COVID-19 sample collection process?	Painless	1	
		Slightly painful	2	
		Did not know	3	
		Moderately painful	4	
		Very painful	5	
	How were you feeling about getting a positive result of COVID-19?	Very scared	1	
		Scared	2	
		Did not know	3	
		Calm	4	
		Very calm	5	
	How afraid were you of losing work if you tested COVID-19 positive?	Very scared	1	
		Scared	2	
		Did not know	3	
		Calm	4	
		Very calm	5	
	What type of information is usually required for the COVID-19 test? INSTRUCTIONS ; Multiple responses are possible	Who was eligible for COVID-19 test?	1	
		When should I get tested for the COVID-19?	2	
		How can I get tested for COVID-19?	3	
		Where are the COVID-19 testing services available near me?	4	
		What is the cost of the COVID-19 test?	5	
		What types of tests are available for COVID-19?	6	
		Which COVID-19 test is most accurate?	7	
		Other (please specify)	99	
	Did you get this information?	Yes	1	►147
		No	2	
	What were the main sources of this information? INSTRUCTIONS: Multiple responses are possible.	Television channels	1	
		Daily newspapers	2	
		Websites or online news pages	3	
		Social media (e.g., Facebook, Twitter, YouTube, WhatsApp)	4	
		Search engines (e.g., Google)	5	
		Conversations with family and friends	6	
		Conversations with colleagues	7	
		Consultation with family physician	8	
		Government campaigns	9	
		Government call centre	10	
		Government hospitals or centres	11	
		Advisories issued by the government	12	
		Elected public representatives	13	
		Civil societies/NGOs	14	
		Other sources (please specify)	99	
	Where were the COVID-19 testing facilities available near you? INSTRUCTIONS: Multiple responses are possible. Yes-1, No-2, Not sure-3	Public sector hospital		
		GOVT. Dispensary/Hospital	1/2/3	
		Urban Community/Primary Health Centre	1/2/3	
		CHC/Block PHC	1/2/3	
		Sub-Centre	1/2/3	
		Health & Wellness Centre	1/2/3	
		Govt. Mobile Clinic	1/2/3	
		Camp	1/2/3	
		Anganwadi/ICDS Centre	1/2/3	
		Asha	1/2/3	
		Other community-based members	1/2/3	
		Other Public Sector Health Facility (please specify)	1/2/3	
		NGO Or Trust Hospital/Clinic	1/2/3	
		Private health sector		
		Private Hospital/ Clinic /Doctor	1/2/3	
		Private laboratories	1/2/3	
		Other private sector health facility (please specify)	1/2/3	
	How far was this COVID-19 testing facility from your house?	Less than 1 Km	1	
		1 to 2 Km	2	
		2 to 3 Km	3	
		3 to 4 Km	4	
		4 to 5 Km	5	
		More than 5 Km	6	
		Do not know	98	
	Was public transport like bus, auto available to reach this COVID-19 testing centre?	Yes	1	
		No	2	
		Do not know	98	
	What was the cost of the COVID-19 test?	It was free in the government sector	1	
		It was not free in government sector	2	
		It was free in private sector	3	
		It was not free in private sector	4	
		It was at a capped price in the private sector	5	
		Do not know	98	
	How easy or difficult the entire process of getting the COVID-19 test was?	Very easy	1	
		Easy	2	
		Neither easy nor difficult	3	
		Difficult	4	
		Very difficult	5	
	What were the chances that you would have got the COVID-19 test done?	Very unlikely	1	
		Unlikely	2	
		Neither likely nor unlikely	3	
		Likely	4	
		Very likely	5	
	When did you decide about the COVID-19 test?	Within one day of developing first symptom	1	
		Second day of developing first symptom	2	
		Third day of developing first symptom	3	
		Fourth day of developing first symptom	4	
		Fifth day of developing first symptom	5	
		Sixth day of developing first symptom	6	
		Seventh day of developing first symptom	7	
		After seventh day of developing first symptom	8	
		Never made a decision	9	
		Decided but don't remember when.	98	
	What was your decision about the COVID-19 test?	Will be tested	1	
		Will not be tested	2	
		Do not know	98	
	What influenced you to make this decision?	Advise of government doctor	1	
		Advise of private doctor	2	
		Health workers advice	3	
		advice from family members	4	
		friends advice	5	
		Past COVID-19 patient advice	6	
		Mass media campaign	7	
		Health campaign	8	
		Nobody	9	
		Other (please specify)	99	
	Did you try for the COVID-19 test?	Yes	1	►158
		No	2	
		Do not know	98	
	What did you try to get the COVID-19 test done? INSTRUCTIONS: Multiple responses are possible.	Visited hospitals/health centres	1	
		Visited COVID-19 testing unit/s	2	
		Visited COVID-19 testing camp/campaign	3	
		Contacted COVID call centre	4	
		Contacted healthcare worker	5	
		Contacted social worker	6	
		Other (please specify)	99	
	Why didn't you try for the COVID-19 test?	I thought I was not sick enough for test	1	
		was advised to isolate myself instead of going for test	2	
		My health condition was not good enough to go to a testing unit	3	
		There was no one to help me reach the testing unit	4	
		I thought the test was useless because there was no treatment for COVID-19	5	
		Other (please specify)	99	
	Were you tested for COVID-19?	Yes	1	►162
		No	2	
		Do not know	98	
	Why haven't you been tested?	I was told that I was not eligible for the test	1	
		I was told that the staff was not available for testing	2	
		I was told that the logistics are not available for testing	3	
		waited for more than five hours and therefore came back home without testing	4	
		There was a lot of crowd at the testing center, so I came back without getting tested	5	
		Other (please specify)	99	
	What did you do after not having a COVID-19 test despite trying or waiting?	Tried again for the test	1	►STOP
		Did not try again for the test	2	
		Others (please specify)	99	
	If you were tested, please let me know when.	The test was done within a day of trying for the test	1	
		The test is done on the second day of trying the test	2	
		The test is done on the third day of trying the test	3	
		The test is done on the fourth day of trying the test	4	
		The test is done on the fifth day of trying the test	5	
		The test is done on the sixth day of trying the test	6	
		The test is done on the seventh day of trying the test	7	
		The test is done after the seventh day of trying the test	8	
		Test done but not remembering when	9	
	Please tell me the waiting time between arrival and sample collection at the COVID-19 testing unit.	One hour	1	
		1 to 2 hours	2	
		2 to 3 hours	3	
		3 to 4 hours	4	
		4 to 5 hours	5	
		More than five hours	6	
	Where were you tested?	Government sector	1	
		Private sector	2	
		Do not know	98	
	Can you tell us which test was done? Instruction: Multiple responses possible	Rapid Antigen Test	1	
		RT-PCR	2	
		CBNAAT	3	
		TrueNat	4	
		Do not know	98	
	Did you pay for COVID-19 test?	No	1	
		Yes	2	
		Do not know	98	
	If yes, please tell the amount in Rs.	Amount in Rs.		
	What was the COVID-19 test result?	Found negative	1	
		Found positive	2	
		Equivocal	3	
		Repeat sample	4	
		Result not received	5	
		Do not know	98	
	When did you get the test result after the sample collection?	Within 6 hours	1	
		Within 6 to 12 hours	2	
		Within 12 to 18 hours	3	
		Within 18 to 24 hours	4	
		Within 24 to 48 hours	5	
		More than 48 hours	6	
		Never	7	
	How did you receive the COVID-19 test result?	From hospital/centre/lab	1	
		Through a call	2	
		Through portal	3	
		Through healthcare worker	4	
		Others (please specify)	99	
	How was the behavior of the staff during the COVID-19 testing?	Very good	1	
		Good	2	
		Neither good nor bad	3	
		Bad	4	
		Very bad	5	
	Were COVID-19 appropriate practices like bearing of the mask, social distancing, and sanitization of hands strictly followed in the testing unit?	Yes	1	
		No	2	
		Cannot say	98	
